# Tech-based evaluation of healthcare quality during the COVID-19 pandemic

**DOI:** 10.1097/MD.0000000000048850

**Published:** 2026-05-22

**Authors:** Kang Wang, Ruixiang Xu, Qian Huang

**Affiliations:** a School of Management, Capital Normal University, Beijing, China; b School of Management, Beijing University of Chinese Medicine, Beijing, China; c Department of Respiratory Medicine, Beijing Tian Tan Hospital, Capital Medical University, Beijing, China.

**Keywords:** COVID-19, Global Burden of Disease, healthcare service quality, machine learning, random forest, support vector machine

## Abstract

The coronavirus disease 2019 pandemic profoundly disrupted global health systems, exacerbating preexisting inequalities in access to and quality of care. Disparities in service accessibility, continuity, coordination, and comprehensiveness highlight the need for a multidimensional, data-driven approach to healthcare evaluation. We conducted a retrospective analysis using 52,490 responses from the 2021 Global Burden of Disease COVID-19 Health Service Disruption Survey. We developed a 4-dimensional evaluation framework aligned with World Health Organization principles for people-centered care. Following variable selection, standardized preprocessing, and manual labeling, we trained and assessed 6 machine learning (ML) algorithms. We further optimized the 2 best-performing models – support vector machine (SVM) and random forest (RF) – using interpolation-based data augmentation, cross-validated hyperparameter tuning, and particle swarm optimization. SVM showed the highest baseline performance (accuracy 0.93), whereas RF achieved competitive macro-F1 scores. Interpolation-based data augmentation improved the generalizability of the SVM model, increasing its accuracy to 0.96. The RF model benefited most from particle swarm optimization-based hyperparameter optimization. Model predictions revealed persistent inequalities in healthcare service quality: respondents with lower income, lower educational attainment, and rural residence were disproportionately classified as receiving lower-quality services, whereas urban residence and higher income were associated with higher-quality care. Our scalable ML framework successfully assessed healthcare service quality during the pandemic, with optimized SVM and RF models demonstrating robust performance on high-dimensional, imbalanced survey data. Post-pandemic recovery strategies must prioritize socioeconomically disadvantaged populations. Furthermore, integrating ML tools can enhance real-time quality monitoring within health systems.

## 1. Introduction

The Global Burden of Disease (GBD) program provides systematic estimates of health loss across 204 countries and territories and serves as an important foundation for evidence-based policymaking.^[1]^ The coronavirus disease 2019 (COVID-19) pandemic placed health systems under exceptional pressure worldwide, disrupting essential services, delaying care, and creating persistent challenges in accessibility, continuity, coordination, and comprehensiveness of care.^[2–5]^ These disruptions were consistently documented in global monitoring reports and pulse surveys conducted by the World Health Organization (WHO).^[2–5]^ The quality of health services is conceptually informed by the structure-process-outcome model of Donabedian and by the core principles of primary care described in analyses from the European Observatory.^[6]^ Coordination and continuity of care, which are central features of people-centered health systems, have been repeatedly associated with better outcomes and lower levels of inequality.^[7]^ WHO frameworks, including integrated people-centered health services (IPCHS) and integrated care for older people (ICOPE), further emphasize the need for service delivery models that ensure accessibility, continuity, comprehensiveness, and responsiveness to population needs.^[8,9]^

High-quality health systems are essential for achieving universal health coverage (UHC), as highlighted by the Lancet Commission on High-Quality Health Systems and other global monitoring initiatives.^[10,11]^ In this context, progress toward UHC depends not only on service availability but also on equitable access and effective coverage, both of which remain unevenly distributed across socioeconomic groups.^[11–13]^ Social determinants of health, including income, education, and geographic location, continue to shape disparities in service use and service quality. These inequities became more visible during the pandemic and underscored the importance of monitoring service restoration.^[14–22]^ At the same time, machine learning (ML) has become a useful approach for analyzing high-dimensional health data and developing scalable evaluation frameworks.^[23–25]^ Although ML has been widely used for diagnostic prediction and risk stratification, its application to system-level assessment of service quality remains limited, particularly in analyses based on globally representative datasets such as the GBD COVID-19 Health Service Disruption Survey.^[26]^ Previous studies using GBD data have mainly focused on disease burden, forecasting models, or effective coverage indices for UHC monitoring.^[1,11,27]^ Multidimensional ML-based evaluation of service disruption and recovery quality remains scarce.

The objective of this study was to build and validate an ML pipeline for the assessment of healthcare service quality across 4 dimensions: accessibility, continuity, coordination, and comprehensiveness, using GBD 2021 survey data. A further objective was to identify disparities in service quality across income groups, regions, and demographic subgroups, thereby providing evidence to support equitable resource allocation and post-pandemic recovery planning.

## 2. Methods

### 2.1. Study design and data source

This study was a retrospective secondary analysis of data from the GBD 2021 COVID-19 Health Service Disruption Survey, a global dataset compiled by the Institute for Health Metrics and Evaluation.^[26]^ The survey included 52,490 responses from populations across different regions and income settings and recorded changes in health service use, reasons for seeking care, medication needs, and chronic disease management during the pandemic. These data complement the WHO global pulse surveys, which also documented widespread disruption to essential health services.^[2–5]^ All records were anonymized and publicly available; therefore, ethical approval was not required.

### 2.2. Four-dimension evaluation framework

Guided by WHO frameworks on IPCHS and UHC monitoring, we developed a 4-dimension analytical framework comprising accessibility, continuity, coordination, and comprehensiveness^[8,11]^ (Table 1). These dimensions parallel foundational concepts in health services research, including the Donabedian model and primary care attributes identified in the European Observatory’s analysis of health systems.^[6,7]^ Accessibility was assessed using indicators of service use before and after the pandemic, care setting, reasons for visits, service coverage radius, and proxy measures of waiting time. Continuity reflected changes in health status and ongoing care needs, in line with earlier WHO ICOPE recommendations.^[9]^ Coordination captured transitions across levels of care and the integration of care networks, which have been associated with improved patient experience and better outcomes.^[7]^ Comprehensiveness included medication needs, treatment indications, and missed doses, which are closely related to continuity in chronic disease management.^[14–19]^ The Service Restoration Index was included as an additional proxy for the extent of recovery from pandemic disruptions, consistent with WHO guidance on maintaining essential services.^[2–5]^

**Table 1:** Variables retained for the 4-dimension framework after screening.

Column name	Meaning
user_id	User account identifier
gp_pre/post_provider_need	Whether the respondent had healthcare service needs before/after the pandemic
gp_pre/post_provider_condition	Reasons for needing healthcare services before/after the pandemic
gp_pre/post_provider_visit	Whether the respondent received needed healthcare services before/after the pandemic
gp_pre/post_provider_where	Level or type of healthcare provider visited before/after the pandemic
gp_pre/post_provider_why	Reasons for not receiving needed healthcare services before/after the pandemic
gp_medication	Medication treatment needs
gp_medication_condition	Reasons for requiring medication treatment
gp_pre/post_miss_dose	Whether the respondent missed medication doses before/after the pandemic
gender/age/educate/labor	Demographic characteristics (gender, age, educational attainment, employment status)
Overall evaluation	Overall evaluation of healthcare service quality

### 2.3. Data selection and preprocessing

**Figure 1.**
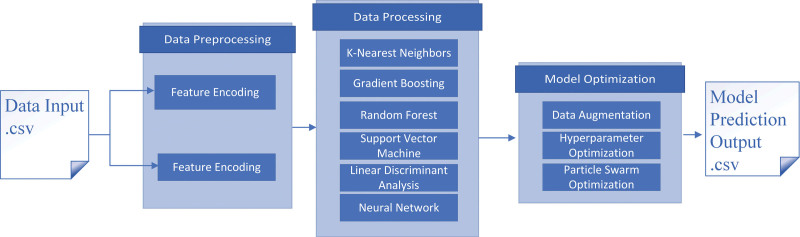
Data processing workflow.

Variables irrelevant to the 4 dimensions were removed, and inconsistent categorical levels were harmonized. Missing values were handled using structured imputation strategies informed by methodological guidance from van Buuren and Little and Rubin.^[28,29]^ Numerical variables were assigned constant placeholders, whereas categorical variables were assigned a “missing” category before 1-hot encoding. Observations without meaningful information related to healthcare or care needs were removed. Feature selection followed widely used criteria described by Chandrashekar et al.^[30]^ All preprocessing steps were implemented in Python using Scikit-learn^[31]^ (Fig. 1).

### 2.4. Manual annotation

To generate ground-truth labels for supervised learning, trained annotators classified the quality of healthcare service received by each respondent as “high,” “moderate,” or “low.” Labeling criteria were derived from WHO quality-of-care standards and UHC monitoring constructs.^[8,9,11,12]^ Approximately 15% of the sample was double-coded, and inter-rater reliability exceeded 90%. Cohen kappa values met the thresholds for “substantial” to “almost perfect” agreement according to published methodological guidelines.^[32,33]^ Disagreements were resolved through consensus meetings to ensure labeling consistency.

### 2.5. Model development and evaluation

Six algorithms commonly used for structured health survey datasets were trained and evaluated: gradient boosting, support vector machine (SVM), random forest (RF), k-nearest neighbors, linear discriminant analysis, and a shallow neural network. The use of these algorithms is supported by previous applications of ML in health research, including predictive analytics and system-level modeling.^[23–25,34–37]^

Data were split into training (70%) and testing (30%) sets. Model performance was evaluated using accuracy, macro-precision, macro-recall, and macro-F1, which provide a balanced assessment in the presence of class imbalance.^[38]^ Confusion matrices were used to examine category-specific misclassifications.

### 2.6. Model optimization

**Figure 2.**
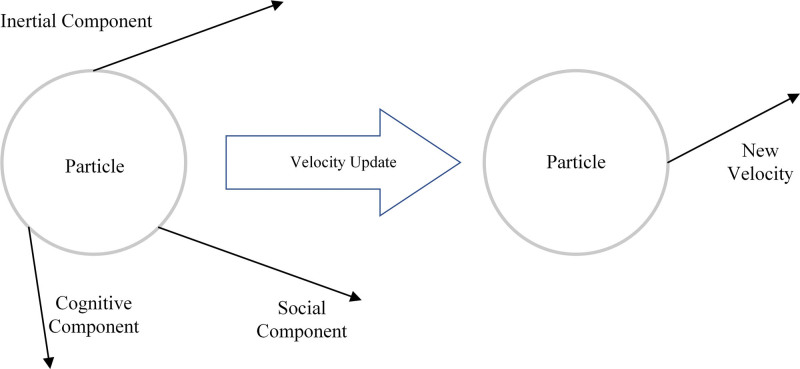
PSO principle and parameter update scheme. PSO = particle swarm optimization.

The 2 best-performing baseline models, SVM and RF, were further optimized through a structured 3-stage procedure. First, interpolation-based augmentation was applied to underrepresented classes using principles similar to those of the Synthetic Minority Over-sampling Technique synthetic sampling framework.^[39,40]^ This step was intended to reduce class imbalance and improve generalizability. Second, hyperparameter tuning was conducted using grid search and random search, both of which have been shown to perform better than manual tuning in high-dimensional settings.^[34,35]^ For SVM, the parameter space included kernel type, C, and γ. For RF, the parameter space included *n_estimators*, *max_depth*, and *min_samples_split*. Third, particle swarm optimization (PSO) was used to explore non-convex parameter spaces. PSO is a population-based metaheuristic that has been widely applied in ML optimization.^[41]^ The optimization process followed canonical velocity-position update rules to improve the efficiency of global search (Fig. 2). Performance metrics followed the scoring convention of Scikit-learn, in which minimization objectives are represented by negated values; these were converted to positive accuracy values for interpretation.^[31]^

### 2.7. Ethical considerations

All data were deidentified and publicly available through the Institute for Health Metrics and Evaluation and the WHO repositories^[26]^. The study followed WHO ethical principles for secondary analysis of health data. No human subjects or animals were directly involved, and institutional review board approval was not required.^[2–5]^

## 3. Results

### 3.1. Baseline model comparison

Among the 6 algorithms evaluated – gradient boosting, SVM, RF, k-nearest neighbors, linear discriminant analysis, and a shallow neural network – SVM showed the best overall baseline performance (Table 2). This is consistent with previous studies reporting that SVM performs well on high-dimensional structured data because of its margin-maximizing properties.^[23,42]^

**Table 2:** Algorithms evaluated and key characteristics with performance summary.

Algorithm	Applicable scenarios	Advantages	Limitations
Gradient boosting	Structured/tabular data; various data types	High performance; capable of handling diverse data types	Complex hyperparameter tuning; higher model complexity
Support vector machine (SVM)	Small-sample, high-dimensional data	Strong generalization ability; suitable for limited datasets	Sensitive to parameter settings; kernel selection can be difficult
Random forest (RF)	High-dimensional data; datasets with many features	Robust performance; handles many features effectively	Longer training time; limited interpretability
k-nearest neighbors (KNN)	Small-scale, low-dimensional data	Simple and intuitive; easy to implement	Low computational efficiency; high memory consumption
Linear discriminant analysis (LDA)	Linearly separable data; small/medium-scale datasets	Computationally simple; effective for linear problems	Strong distributional assumptions; low flexibility
Neural network	Large-scale datasets; complex pattern recognition	Strong nonlinear modeling ability; highly adaptable	Difficult to train; prone to overfitting

**Figure 3.**
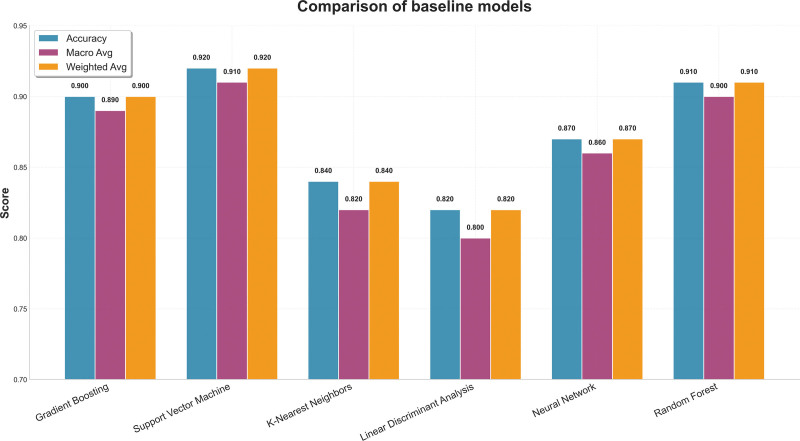
Comparison of baseline models (accuracy, precision/recall/F1).

The SVM achieved an accuracy of 0.93 and a weighted F1-score of 0.93. RF also performed well, with a macro-F1 score of 0.91, in line with earlier reports that ensemble methods are effective for tabular classification tasks.^[43]^ Gradient boosting also achieved good accuracy, although its performance varied more across folds. This may reflect sensitivity to heterogeneous categorical encoding and class imbalance, which are known challenges in this type of dataset^[36,38]^ (Fig. 3).

### 3.2. Optimization outcomes

#### 3.2.1. Support vector machine

Interpolation-based augmentation improved the generalization of SVM. Synthetic minority sampling, using a strategy similar to Synthetic Minority Over-sampling Technique, improved class balance and produced the highest test accuracy of 0.9644.^[39,40]^ This is consistent with previous studies showing that resampling can significantly improve classifier robustness in imbalanced datasets.^[38]^

**Figure 4.**
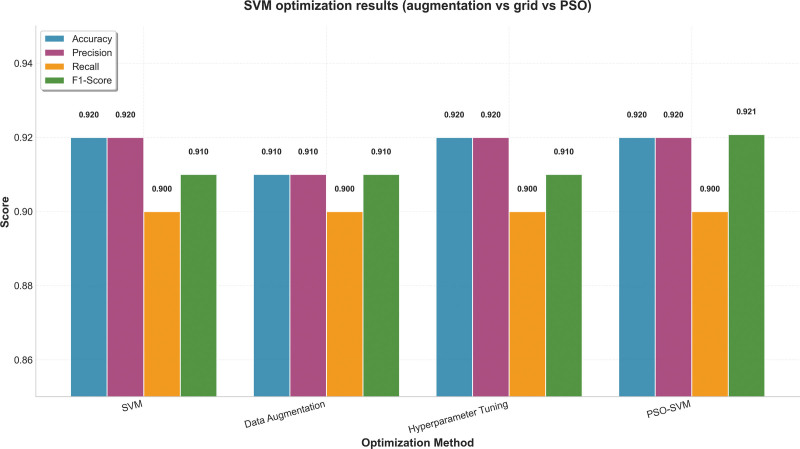
SVM optimization results (augmentation vs grid vs PSO). PSO = particle swarm optimization, SVM = support vector machine.

Additional hyperparameter tuning with grid search and random search led to only small additional gains. This agrees with previous methodological studies suggesting that SVM performance is mainly determined by kernel selection and margin-related parameters.^[34,35]^ PSO produced little further improvement, possibly because the SVM parameter space is less dependent on broad global exploration than that of tree-based models^[41]^ (Fig. 4).

#### 3.2.2. Random forest

For RF, PSO produced the largest performance improvement, outperforming both grid search and random search. Its population-based search may better capture complex interactions among parameters such as tree depth, split thresholds, and the number of estimators, all of which strongly affect RF performance.^[41]^ RF accuracy improved to 0.931 after optimization.^[31]^

**Figure 5.**
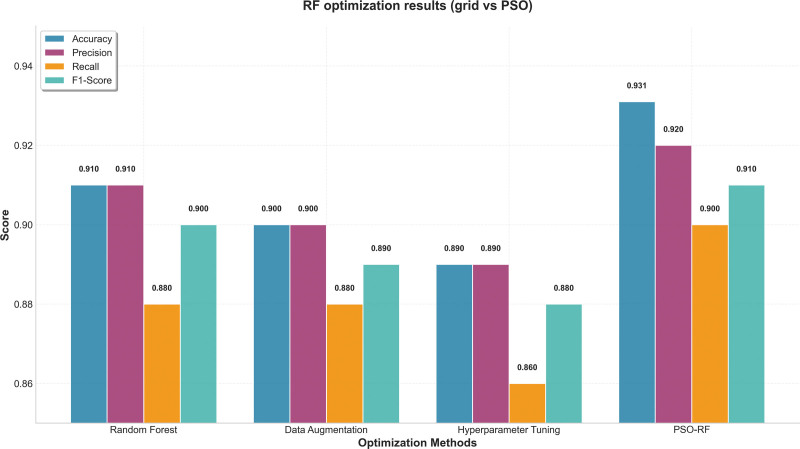
RF optimization results (grid vs PSO). PSO = particle swarm optimization, RF = random forest.

Data augmentation also improved RF performance, but the gain was smaller than that achieved through PSO-based hyperparameter optimization. This is consistent with previous work suggesting that ensemble models often benefit more from structural parameter tuning than from synthetic resampling alone^[36,37]^ (Fig. 5).

### 3.3. Disparities in predicted service quality

**Figure 6.**
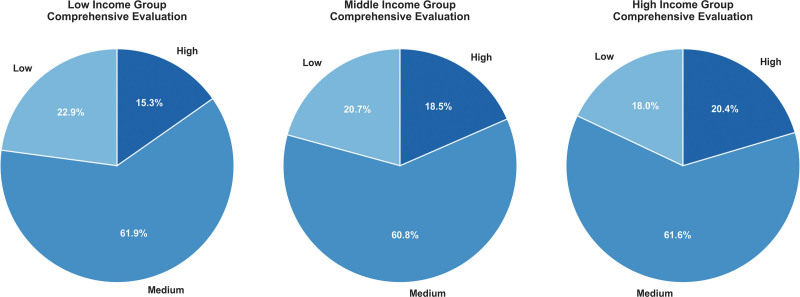
Distribution of predicted service levels by income group.

Model predictions showed clear socioeconomic disparities in estimated healthcare service quality. Respondents in lower-income groups were more often classified as receiving “low” quality services, whereas those in higher-income and urban groups were more frequently classified as receiving “high” quality care. These findings are consistent with the WHO global UHC monitoring report and previous studies on the social determinants of health^[1,11,27]^ (Fig. 6).

Rural residents exhibited significantly lower accessibility and continuity scores, consistent with long-standing evidence showing barriers faced by rural populations in low- and middle-income countries.^[14–16,18,19]^ In contrast, urban residents and respondents with higher education demonstrated more stable continuity and better coordination patterns, reflecting stronger integration between primary and referral care services.^[6,7]^

Age-related differences were also observed. Older adults had lower continuity and coordination scores, which may be related to deferred care, multimorbidity, and disruptions in chronic disease management during the pandemic.^[9,14–18]^ These findings are also consistent with the WHO ICOPE framework, which highlights the vulnerability of older populations during health system shocks.

**Figure 7.**
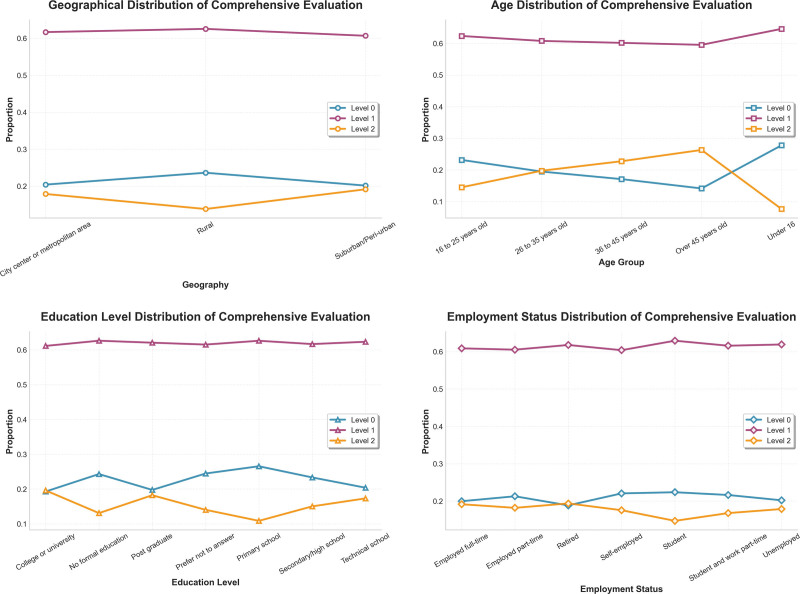
Regional and socioeconomic correlates of predicted service quality.

Regional disparities also followed global income patterns, in agreement with findings from the GBD Healthcare Access and Quality Index and UHC effective coverage analyses.^[27]^ Higher-income regions displayed stronger recovery of health service delivery, while lower-income regions continued to experience gaps in access and comprehensiveness (Fig. 7).

## 4. Discussion

This study presents a scalable ML framework for the multidimensional assessment of healthcare service quality during the COVID-19 pandemic. By integrating WHO people-centered care domains and leveraging a large global dataset, the framework quantifies accessibility, continuity, coordination, and comprehensiveness across diverse population groups. Our findings yield 2 main insights related to methodological performance and health system disparities.

First, the results indicate that both model selection and optimization strategy substantially influence predictive performance. Data augmentation markedly improved SVM performance by reducing class imbalance, a well-recognized challenge in health survey datasets.^[38–40]^ The optimized SVM model achieved an accuracy of 0.9644, supporting previous evidence that SVM maintains strong generalizability in high-dimensional settings.^[23,42]^ We speculate that the robust performance of the SVM model in this context is due to its ability to define clear decision boundaries even when survey responses present sparse or overlapping feature spaces.

By contrast, RF benefited most from PSO-based hyperparameter optimization. PSO enabled more effective exploration of non-convex parameter spaces, improving RF accuracy beyond that achieved with conventional grid or random search.^[41]^ This pattern is consistent with comparative ML studies showing that tree-based ensemble methods are highly responsive to structural hyperparameter tuning, whereas margin-based models such as SVM rely more on data balancing and kernel selection.^[36,37]^

Taken together, these findings reinforce that no single optimization strategy is universally superior; rather, optimal performance depends on the underlying model architecture. This observation is consistent with the broader ML literature, which supports tailored, model-specific optimization strategies to maximize predictive performance.^[23–25,34–37]^

Second, our results underscore the persistence of socioeconomic inequities in healthcare service quality during pandemic recovery. Respondents with low income, low educational attainment, and rural residence were consistently classified into lower service-quality categories, reflecting long-standing evidence on social determinants of health and resource disparities.^[12,13]^ These gradients also reflect patterns reported in WHO UHC monitoring and GBD effective coverage analyses, where poorer regions exhibit systematically lower accessibility and continuity.^[1,11,27]^ It is highly probable that the pandemic exacerbated preexisting structural weaknesses in these areas, turning chronic underfunding into acute service failures.

Urban residents and higher-income groups showed significantly better predicted service levels, indicating more resilient access to care during periods of disruption. Similar findings have been reported in global studies documenting service delays, deferred care, and reduced healthcare utilization during COVID-19, with disproportionately greater effects among socioeconomically disadvantaged groups.^[14–22]^ We hypothesize that the resilience observed in higher-income and urban cohorts was largely driven by the rapid digital pivot to telemedicine, as well as the financial capacity to bypass overwhelmed public facilities in favor of private care – options largely inaccessible to rural and low-income populations. These disparities highlight persistent obstacles to achieving equitable, people-centered health systems, as envisioned by WHO’s IPCHS framework.^[7]^

### 4.1. Policy and practice implications

The ML-based evaluation framework developed in this study has several implications for health system monitoring and policy planning. By aligning with WHO’s IPCHS model and UHC monitoring approach, the framework could be incorporated into national and regional surveillance systems to track the restoration of essential services and identify emerging inequities.^[8,11]^ The reproducibility and interpretability of the analytic pipeline also support its integration into decision-support tools for resource allocation, including rural outreach, chronic disease follow-up, and stabilization of medication supply chains. These applications are directly aligned with the WHO operational guidance for maintaining essential services during public health emergencies.^[2–5]^

The findings further indicate that pandemic recovery has been uneven, mirroring earlier analyses showing that poorer and rural populations experienced larger service deficits. Targeted interventions, including community-based care coordination, mobile service delivery, and digital health support, are therefore needed to restore accessibility, continuity, and comprehensiveness for underserved groups. The observed age-related disparities also suggest that older adults should be prioritized within integrated care pathways, in line with WHO ICOPE recommendations.^[9]^

### 4.2. Methodological strengths and limitations

This study has several strengths. First, it uses a large, globally representative dataset, the GBD 2021 COVID-19 Health Service Disruption Survey, supplemented by earlier WHO pulse surveys documenting pandemic-related service interruptions.^[2–5]^ Second, the analytic pipeline incorporates systematic preprocessing, structured feature selection based on established ML methodologies, and highly reliable manual labeling supported by robust inter-rater reliability metrics.^[30,32,33]^ Third, the comprehensive comparison of 6 algorithms, followed by model-specific optimization using augmentation, grid/random search, and PSO, provides a rigorous and reproducible workflow aligned with best practices in ML research.^[31,34–37,41]^

Several limitations should also be acknowledged. First, the dataset is observational and self-reported and is therefore susceptible to recall bias and social desirability bias.^[44]^ Second, although manual annotation showed high reliability, it reflects operationalized quality constructs rather than objective service outcomes. Third, residual confounding may remain, particularly for socioeconomic and regional factors not captured in the survey. Fourth, external validation using independent post-pandemic datasets is required to assess generalizability beyond GBD survey respondents. Future research could incorporate causal inference methods, transfer learning, or Bayesian model averaging to enhance interpretability and robustness.

### 4.3. Comparison with existing literature

Our findings extend previous ML applications in healthcare, which have largely focused on diagnostic prediction or risk stratification rather than system-level assessment of service quality. While traditional epidemiological evaluations of survey data heavily rely on generalized linear models to identify associations, our ML pipeline offers a comparative advantage. For instance, whereas recent global assessments, such as those by Gertz et al,^[45]^ utilized multivariable logistic regression to identify socioeconomic predictors of forgone care during the pandemic, our optimized SVM and RF models successfully capture complex, nonlinear interactions among demographic variables and service disruption patterns without relying on strict assumptions of data distribution. Prior studies have shown that SVM and RF perform well in high-dimensional clinical datasets, which is consistent with our performance results.^[42,43]^

Moreover, extensive literature shows that the COVID-19 pandemic caused significant reductions in routine care, emergency visits, vaccinations, and chronic disease management.^[14–19]^ The socioeconomic and regional disparities identified by our models echo global evaluations of excess mortality, deferred care, and disrupted essential health services.^[20–22,46]^ Furthermore, our hypothesis regarding the resilience of urban and high-income cohorts is directly supported by comparative studies like that of Hsiao et al.^[47]^ They demonstrated that the rapid structural shift toward telemedicine during lockdowns disproportionately benefited well-resourced populations with high digital literacy, inadvertently widening the healthcare divide for rural and low-income groups. By moving beyond traditional descriptive statistics, our framework provides a more granular, predictive map of where and for whom these systems failed most critically. By integrating GBD survey data with ML methods, this study makes a novel contribution by moving beyond descriptive analysis to enable multidimensional, respondent-level classification of service quality. This approach is consistent with emerging recommendations to modernize health system performance assessment.^[10,27,48]^

## 5. Conclusion

In summary, this study provides a 4-dimensional, ML-enabled evaluation of healthcare service quality during COVID-19 disruptions using a globally representative dataset. The optimized SVM and PSO-enhanced RF models achieved the strongest predictive performance, demonstrating complementary strengths for the analysis of complex, imbalanced health-service data. The analysis reveals substantial socioeconomic and regional inequities in service restoration, highlighting the need for targeted interventions to support disadvantaged groups. By aligning with WHO’s people-centered and UHC frameworks, the proposed pipeline offers a scalable foundation for continuous monitoring and more equitable post-pandemic health system recovery.^[8,11]^

## Acknowledgments

The authors gratefully acknowledge the Institute for Health Metrics and Evaluation (IHME) for providing access to the publicly available 2021 Global Burden of Disease COVID-19 Health Service Disruption Survey dataset, and the World Health Organization for making available the relevant frameworks, guidance documents, and global pulse survey reports that informed the conceptual development of this study. The authors also acknowledge the support of Capital Normal University, Beijing University of Chinese Medicine, and Beijing Tian Tan Hospital, Capital Medical University, for their academic and institutional support.

## Author contributions

**Conceptualization:** Kang Wang.

**Methodology:** Qian Huang.

**Data curation:** Ruixiang Xu.

**Formal analysis:** Kang Wang.

**Software:** Kang Wang, Ruixiang Xu.

**Visualization:** Ruixiang Xu.

**Writing – original draft:** Kang Wang, Ruixiang Xu.

**Writing – review & editing:** Qian Huang.
